# Air sampling procedures to evaluate microbial contamination: a comparison between active and passive methods in operating theatres

**DOI:** 10.1186/1471-2458-12-594

**Published:** 2012-08-02

**Authors:** Christian Napoli, Vincenzo Marcotrigiano, Maria Teresa Montagna

**Affiliations:** 1 Department of Biomedical Sciences and Human Oncology, University of Bari Aldo Moro, Piazza G. Cesare, 11, 70124, Bari, Italy; 2 Prevention Department, Local Social Health Unit n 7, Via Lubin, 16, 31053, Pieve di Soligo (TV), Italy

**Keywords:** Bioaerosol, Air sampling, Operating theatres, Surveillance

## Abstract

**Background:**

Since air can play a central role as a reservoir for microorganisms, in controlled environments such as operating theatres regular microbial monitoring is useful to measure air quality and identify critical situations. The aim of this study is to assess microbial contamination levels in operating theatres using both an active and a passive sampling method and then to assess if there is a correlation between the results of the two different sampling methods.

**Methods:**

The study was performed in 32 turbulent air flow operating theatres of a University Hospital in Southern Italy. Active sampling was carried out using the Surface Air System and passive sampling with settle plates, in accordance with ISO 14698. The Total Viable Count (TVC) was evaluated *at rest* (in the morning before the beginning of surgical activity) and *in operational* (during surgery).

**Results:**

The mean TVC *at rest* was 12.4 CFU/m^3^ and 722.5 CFU/m^2^/h for active and passive samplings respectively. The mean *in operational* TVC was 93.8 CFU/m^3^ (SD = 52.69; range = 22-256) and 10496.5 CFU/m^2^/h (SD = 7460.5; range = 1415.5-25479.7) for active and passive samplings respectively. Statistical analysis confirmed that the two methods correlate in a comparable way with the quality of air.

**Conclusion:**

It is possible to conclude that both methods can be used for general monitoring of air contamination, such as routine surveillance programs. However, the choice must be made between one or the other to obtain specific information.

## Background

Microorganisms that cause infections in healthcare facilities include bacteria, fungi and viruses and are commonly found in the patient’s own endogenous flora, but can also originate from health care personnel and from environmental sources [1]. In particular, the environmental matrices (water, air and surfaces) play a leading role as reservoirs of microorganisms [1]: e.g. *Legionella* spp. and *Pseudomonas aeruginosa* are often isolated from water samples in hospital facilities [2,3]; influenza A virus and other viruses from air [4]; spores of filamentous fungi from surfaces in operating theatres [5]. For this reason, hospital environmental control procedures can be an effective support in reducing nosocomial infections [1,6,7]. This is particularly true in high risk healthcare departments where patients are more susceptible because of their health conditions, or in operating theatres because of tissue exposure to air [8-10]. In fact, surgeons were the first to deal with environmental hygiene conditions during high risk surgery in order to reduce post-operative infections [11,12]. Since then, many authors have underlined the importance of microbial surveillance of environmental matrices [1,2,5,13-15].

A special focus has been placed on microbial air surveillance; in fact, it has been demonstrated that periprosthetic infection rates correlate with the number of airborne bacteria within the wound [16] and that, in hospital environments, the use of air filtration through a HEPA system completely eliminated invasive pulmonary aspergillosis in immune-compromised patients [17].

Through air sampling, it is possible to evaluate microbial contamination in environments at high risk of infection. Moreover, these controls can be used to check the efficiency of both the Conditioned and Controlled Ventilation System (CCVS) and the team’s hygiene procedures. However, although there is much published research, procedures have not been firmly established and there is still debate on the sampling techniques to be used, their frequency of application and even on the usefulness of such checks and controls [18]. In fact, international standards offer different techniques (active or passive sampling) and different kinds of samplers, thus leaving the choice of system open [18,19].

In active monitoring a microbiological air sampler physically draws a known volume of air through or over a particle collection device which can be a liquid or a solid culture media or a nitrocellulose membrane and the quantity of microorganisms present is measured in CFU (colony forming units)/m^3^ of air. This system is applicable when the concentration of microorganisms is not very high, such as in an operating theatre and other hospital controlled environments [18-21].

Passive monitoring uses “settle plates”, which are standard Petri dishes containing culture media, which are exposed to the air for a given time in order to collect biological particles which “sediment” out and are then incubated. Results are expressed in CFU/plate/time or in CFU/m^2^/hour [22]. According to some authors, passive sampling provides a valid risk assessment as it measures the harmful part of the airborne population which falls onto a critical surface, such as in the surgical cut or on the instruments in operating theatres [23].

Several studies have attempted to compare the values of microbial loads obtained through both active and passive samplings, but with inconsistent results: in some cases there was significant correlation [24-26] while in others there was none [27,28]. Currently, since air sampling protocols are not standardized, it is difficult to compare results from different studies [18]. In fact, it has been known for some time that different active samplers show high variability giving different results in the same place at the same time [18]. Whyte found a correlation between the active and passive method, comparing settle plates with the *Active Casella Slit Sampler*[24], while Sayer et al. did not find this correlation using the *Andersen Active Sampler*[28], and Petti et al. demonstrated that, at low air contamination levels, results provided by active *Surface Air System sampler* (SAS) and settle plates were not correlated [21]. Sampling was also carried out in different places in the different studies: Whyte studied the clean-room of a pharmaceutical company, while Petti et al. analysed Dentists’ outpatients clinics. Different indoor environments have different levels of bio-contamination, different kinds of airflow, different numbers of people working in them who use different kinds of personal protective equipment, all factors which affect the results of both the sampling and the comparison between methods [18,22]. Sampling can also be carried out in different moments: Perdelli et al. compared the SAS with the Index of Microbial Air Contamination (IMA) during the surgical activity (*in operational)* when contamination is higher. Additionally, it could be interesting to also study the bio-contamination before the start of the operation (at rest) when the room is empty, as the ISO norm suggests, in this way checking the performance capabilities of the theatre, especially its air systems [19].

Given this research background it is of fundamental importance that researches continue in order to investigate if there is a real correlation between the two methods, between the results provided by different samplers and in different indoor environments, so using scientific evidence to eventually lead to the proposal of a fixed standard protocol for a correct surveillance procedure.

The aim of the present study is to contribute to the scientific evidence of the previous studies through a comparison between two of the widely used methods (active SAS and passive IMA) in the operating theatres of one hospital in Southern Italy. Bio-contamination surveillance was carried with both methods, to be compared later, at the two moments suggested by the ISO norm: *at rest* and *in operational* with a standardized protocol.

## Methods

The study was carried out in the largest hospital of the Apulia Region in South-eastern Italy which is composed up of 32 separate buildings with 60 bed-operating units, for a total bed capacity of 1400, and with an average number of surgical operations greater than 120/day. Thirty-two turbulent air flow operating theatres within 13 surgical departments were enrolled; at the time of sampling, all operating rooms were equipped with HEPA filters. The mean room volume was 136.9 m^3^ (SD: ± 15.2; range = 112.1-158.7). Sampling was performed between September-October 2010.

Following the study protocol, air from one operating room per day was sampled with both active and passive methods at the same time. In each room sampling was performed *at rest* (in the early morning before the beginning of surgical activity) and *in operational* (during surgery). In addition, the number of personnel present *in operational* was recorded to assess the association between the number of people in the room and the value of Total Viable Count (TVC). The sampling staff took great care in hand and forearm washing and in accurate use of personal protective equipment such as gowns, masks, caps, gloves and overshoes.

### Passive sampling

Passive sampling was performed to determine the *Index of Microbial Air Contamination* (IMA) [22]. This index corresponds to the number of CFU counted on a Petri dish with a diameter of 9 cm placed according to the 1/1/1 scheme (for 1 hour, 1 m above the floor, about 1 m away from walls or any major obstacles). In our study the IMA plates (one for TVC and one for filamentous fungi) were placed in the operating theatre approximately 1 m from the operating table, with results expressed in CFU/m^2^/h. Since no standard limits for IMA are provided by Italian official documents, the Swiss Hospital Association standards were considered as maximum levels of IMA in operating theatres with turbulent air flow: ≤786.4 CFU/m^2^/h (≤5 CFU/9 cm diameter plate/h) *at rest*, and ≤3932.1 CFU/m^2^/h (≤25 CFU/9 cm diameter plate/h) *in operational*[29].

### Active sampling

All active sampling was performed using the same Surface Air System Sampler (SAS, International PBI, Milan, Italy), with a flow rate of 180 L/min. The sampler was placed immediately beside the IMA plates.

Both the Italian Institute for Occupational Safety and Prevention (ISPESL) and the International Standard Organization (ISO), in their official documents for bio-contamination control in operating rooms, do not provide precise recommendations with regard to the sampling protocol (precise air volume to be sampled, length of sampling time etc.) [19,30]. As reported by Pasquarella et al., a volume of 500 L of air was sampled *at rest* in one continuous drawing [3], because *at rest,* when the room is empty of people, the results of the sampling reflect mainly the performance of the CCVS [18,19]; in this situation, a single continuous drawing can be comparable to one hour of settle plates exposure.

During *in operational* sampling, when the personnel is in the room, the results of the sampling clearly reflect the team’s hygiene procedures and behaviour, and not only the CCVS performance [18,19]. For this reason, active sampling was carried out over the period of the hour that the IMA plates were exposed, with 5 separate air draws of 100 L each for a total volume of 500 L, with intervals of 12 minutes between draws. In fact, Perdelli et al. found that a correlation between the two methods is possible when the active sampling is carried out at regular intervals during the exposure time of the settle plate [26], because a single drawing detects the contamination only during the short time necessary for the drawing and is therefore not able to detect what the IMA plate detected over the complete hour. Even the ISPESL guidelines suggest, only *in operational,* an active serial sampling carried out at regular intervals [30].

The number of CFUs was adjusted using the conversion table provided by the manufacturer, and the value was expressed in CFU/m^3^. Maximum acceptable levels were taken as the standards determined by ISPESL in 2009 for air microbial contamination in operating theatres with turbulent air flow: ≤ 35 CFU/m^3^*at rest* and ≤ 180 CFU/m^3^*in operational*[30].

### Laboratory methods

For both IMA and SAS, TVC was recorded using Tryptic Soy Agar (TSA), with plates incubated at a mean temperature of 36 ± 1°C for 48 h. Presence of filamentous fungi was also evaluated using plates containing *Sabouraud chloramphenicol dextrose agar* (SabC, Becton-Dickinson, Heidelberg, Germany), incubated at 30°C for 10 days and identified on the basis of their macroscopic and microscopic morphological features [31].

All laboratory tests were carried out at the “Hygiene” Operating Unit (Quality certified according to standard ISO 9001:2008), at the University Hospital “Policlinico Consorziale”, Bari, Italy.

### Statistical analysis

The results from the two sampling methods were loaded into a database created with the software *File Maker* and data analysis was performed using SPSS vs. 16.0 software (IBM Corporation, New York, US). To assess the correlation between the results obtained through the two different sampling methods, both *at rest* and *in operational*, Spearman’s rank correlation coefficient (significance α level was established at 0.05) and a linear regression model were used. In addition, linear regression was used to analyse the relationship between the number of people present in the operating room and the bacterial loads for each method. A p-value of <0.05 was regarded as significant in the linear regression analysis.

## Results and Discussion

The number of samplings, for each of the active and passive methods, was 32 *at rest* and 19 *in operational*, as in the other 13 rooms no surgical activities followed sampling *at rest*.

The mean TVC *at rest* was 12.4 CFU/m^3^ (SD = 12.1; range = 0-56) and 722.5 CFU/m^2^/h (SD = 1035.5; range = 0-4718.5) for active and passive samplings respectively.

The mean *in operational* TVC was 93.8 CFU/m^3^ (SD = 52.69; range = 22-256) and 10496.5 CFU/m^2^/h (SD = 7460.5; range = 1415.5-25479.7) for active and passive samplings respectively.

Fungi were isolated only during two separate surgical operations: in the first IMA allowed the identification of a colony of *Aspergillus* spp*.* and in the second SAS revealed the presence of *Penicillium* spp*.*

*At rest*, 1 (3.1%) and 7 (21.9%) samples exceeded the limit value of the active (35 CFU/m^3^) and of the passive method (786.4 CFU/m^2^/h) respectively. With *in operational* sampling, 1 (5.3%) and 14 (73.7%) samples exceeded the limit value of the active (180 CFU/m^3^) and of the passive method (3932.1 CFU/m^2^/h) respectively.

**Figure 1:**
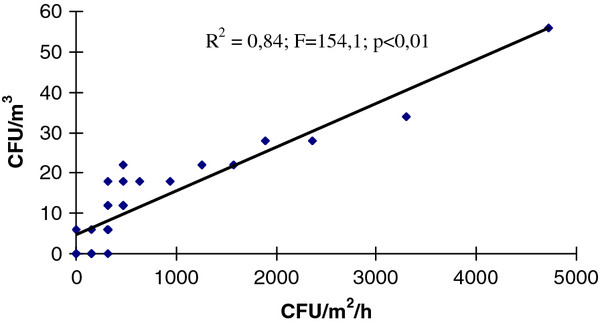
**Correlation between the TVC values detected simultaneously by IMA (CFU/m**^**2**^**/h) and SAS (CFU/m**^**3**^**) in 32 operating rooms* at rest.***

**Figure 2:**
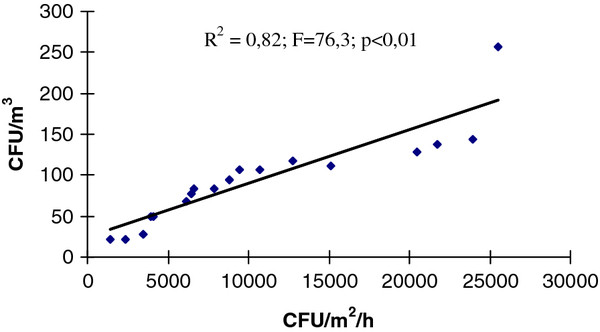
**Correlation between the TVC values detected simultaneously by IMA (CFU/m**^**2**^**/h) and SAS (CFU/m**^**3**^**) in 19 operating rooms* in operational. ***

The Spearman’s test shows in both sampling moments (*at rest* and *in operational*), the high correlation between the results of the two sampling techniques (r_s-before_ = 0.96; r_s-during_ = 0.99): when CFU/m^3^ grew the IMA also grew (α < 0.05). The correlation between methods at rest (R^2^ = 0.84; F = 154.1; p < 0.01) and in operational (R^2^ = 0.82; F = 76.3; p < 0.01) was also demonstrated by the regression model (Figures 1 and 2).

*In operational* sampling showed higher values of TVC than *at rest* with both active and passive methods (93.8 vs 12.4 CFU/m^3^ and 10496.5 vs 722.5 CFU/m^2^/h respectively) as would be expected due to the inevitable microbial dispersion from people. Linear regression, in fact, revealed a significant association between the number of people and the TVC with both methods: IMA (R^2^ = 0.610; F = 26.3; p < 0.01) and SAS (R^2^ = 0.608; F = 26.6; p < 0.01). The mean number of people present in the operating theatre during the 19 *in operational* samplings was high at 7.4 (SD = 3.1; range = 3-13). This is typical of university hospitals in Italy where teaching is done directly in the theatre.

A study published in 2012 found that levels of recorded microbial contamination in operating rooms are also influenced by external factors such as the point of collection in the operating room [32]; so confirming previous reports in which, with the passive sampling method, higher counts were found on settle plates nearer the wound than in periphery [33]. Our study investigated only one sampling point located 1 m away from the surgical table (as recommended by the guidelines) and, in this position, 14 of the 19 passive samples exceeded the limit value. In the light of the 2012 study, sampling near the wound would have probably resulted in all plates being over the limit, showing that the situation is even more critical.

With regard to fungi contamination, only two different strains of mould were identified, one by IMA and one by SAS. These results are in accordance with those of two previous studies carried out in controlled environments of the same hospital, where an uncommon fungi contamination was found [34,35]. Our data do not confirm the findings from Verhoeff et al., which showed that active sampling was better at collecting fungal species [36] and from Asefa et al. which found that the SAS air sampler showed higher numbers of fungi species and mean CFU/plate compared to settle plates [37]. However, the operating rooms in our study were equipped with HEPA filters unlike indoor environments in the studies of Verhoeff et al. and Asefa et al. Other authors have reported that fungal air contamination was never detected in rooms equipped with HEPA filters [38,39] and that simple protective measures, such as air filtration, are known to be effective against mould complications in hospitalized patients [17].

## Conclusions

The microbiological quality of the air in operating theatres is a significant parameter to control healthcare associated infections, and regular microbial monitoring can represent an useful tool to assess environmental quality and to identify critical situations which require corrective intervention. The microbiological content of the air can be monitored by two main methods, one active and one passive. However, at the moment, there are no specific indications with regard to the protocol to be used in air sampling, neither in the Italian ISPESL guidelines, nor internationally in the ISO standards. This has created a strange situation in that there are recommended target limits, such as the ones provided by ISPESL, but no precise guidelines on how to obtain the TVC value. Moreover, previous studies have not given consistent results due to the different samplers used, the different places sampled (operating rooms, dental clinics, pharmaceutical clean-rooms etc.) and/or the different parameters applied (volume of air sampled, sampling time protocol, point of sampling, etc.).

Our study has demonstrated that when a strict protocol is followed results of active and passive sampling correlate in a comparable way with the quality of air for both *at rest* and *in operational* sampling. Further studies must now be undertaken to confirm this result.

In the meantime, it is possible to conclude that both methods can be used for general monitoring of air contamination, such as routine surveillance programs. However, the choice must be made between one or the other to obtain specific information. In particular, if the air sampling performed during surgery is carried out to monitor the risk of microbial wound contamination, passive measurement is better than volumetric sampling at predicting the likely contamination rate at the surgical site, as it allows a direct measure of the number of microorganism settling on surfaces [19,40,41]. On the contrary, if the sampling is performed to obtain information on the concentration of all inhalable viable particles, the active method should be preferred [19].

## Competing interests

The authors declare that they have no competing interests.

## Authors’ contribution

CN contributed to the definition of the study protocol, to the data collection, input and analysis and to the manuscript drafting and writing; VM contributed to the data collection, input and analysis and to the manuscript drafting and writing; MTM contributed to the data collection, input and to the manuscript drafting and writing. All authors read and approved the final manuscript.

## Pre-publication history

The pre-publication history for this paper can be accessed here:

http://www.biomedcentral.com/1471-2458/12/594/prepub
